# NH_4_^+^ protects tomato plants against *Pseudomonas syringae* by activation of systemic acquired acclimation

**DOI:** 10.1093/jxb/erv382

**Published:** 2015-08-05

**Authors:** Emma Fernández-Crespo, Loredana Scalschi, Eugenio Llorens, Pilar García-Agustín, Gemma Camañes

**Affiliations:** Grupo de Bioquímica y Biotecnología, Área de Fisiología Vegetal, Departamento de Ciencias Agrarias y del Medio Natural, ESTCE. Universitat Jaume I, 12071 Castellón, Spain

**Keywords:** ABA, H_2_O_2_, induced resistance, NH_4_^+^ nutrition, putrescine, systemic acquired acclimation.

## Abstract

NH_4_^+^ nutrition provokes mild toxicity by enhancing H_2_O_2_ accumulation, which acts as a signal activating systemic acquired acclimation (SAA). Until now, induced resistance mechanisms in response to an abiotic stimulus and related to SAA were only reported for exposure to a subsequent abiotic stress. Herein, the first evidence is provided that this acclimation to an abiotic stimulus induces resistance to later pathogen infection, since NH_4_^+^ nutrition (N-NH_4_^+^)-induced resistance (NH_4_^+^-IR) against *Pseudomonas syringae* pv tomato DC3000 (*Pst*) in tomato plants was demonstrated. N-NH_4_^+^ plants displayed basal H_2_O_2_, abscisic acid (ABA), and putrescine (Put) accumulation. H_2_O_2_ accumulation acted as a signal to induce ABA-dependent signalling pathways required to prevent NH_4_^+^ toxicity. This acclimatory event provoked an increase in resistance against later pathogen infection. N-NH_4_^+^ plants displayed basal stomatal closure produced by H_2_O_2_ derived from enhanced *CuAO* and *rboh1* activity that may reduce the entry of bacteria into the mesophyll, diminishing the disease symptoms as well as strongly inducing the oxidative burst upon *Pst* infection, favouring NH_4_^+^-IR. Experiments with inhibitors of Put accumulation and the ABA-deficient mutant *flacca* demonstrated that Put and ABA downstream signalling pathways are required to complete NH_4_^+^-IR. The metabolic profile revealed that infected N-NH_4_^+^ plants showed greater ferulic acid accumulation compared with control plants. Although classical salicylic acid (SA)-dependent responses against biotrophic pathogens were not found, the important role of Put in the resistance of tomato against *Pst* was demonstrated. Moreover, this work revealed the cross-talk between abiotic stress acclimation (NH_4_^+^ nutrition) and resistance to subsequent *Pst* infection.

## Highlight

NH_4_^+^ protects tomato plants against *Pseudomonas syringae* by triggering putrescine, ABA, and H_2_O_2_ accumulation. H_2_O_2_ derived from NH_4_^+^ mild chronic stress induces ABA-dependent signalling pathways required for the establishment of systemic acquired acclimation.

## Introduction

Plants employ diverse constitutive and inducible defence strategies to counteract colonization by microbial pathogens (Spoel and Dong, 2012). One of the earliest cellular responses following elicitation of pathogen-associated molecular patterns (PAMPs) is the oxidative burst produced by NADPH oxidases, cell wall peroxidases, or polyamine oxidases (Yoda *et al.*, 2006). In addition to the classical salicylic acid (SA) and jasmonic acid (JA)/ethylene defence pathways, plant immunity to microbial pathogens is regulated by distinct pathways related to nitrogen (N) compounds such as amino acids and polyamines (PAs) (Takahashi and Kakehi, 2010; Zeier, 2013). PAs, including putrescine (Put), spermidine (Spd), and spermine (Spm), are positively charged small metabolites implicated in physiological processes, including organogenesis, embryogenesis, floral initiation and development, leaf senescence, pollen tube growth, and fruit development and ripening (Tiburcio *et al.*, 2014). PAs are synthesized from amino acids by decarboxylation of ornithine or arginine by ornithine decarboxylase (ODC) or arginine decarboxylase (ADC), respectively (Walters, 2003). In addition to free PAs, some PAs are conjugated to hydroxycinnamic acids, and the products of PA oxidation participate in the response to abiotic and biotic stresses (Tiburcio *et al.*, 2014). Oxidation of PAs by copper amine oxidases (CuAOs) contributes to the regulation of PA homeostasis and generates catabolic products with biological functions. CuAOs are homodimeric enzymes with high affinity for oxidizing the primary amino groups of Put and cadaverine, and lower affinity for Spd and Spm (Moschou *et al.*, 2012). It is commonly accepted that H_2_O_2_ produced by CuAO in Put oxidation has an important role in stress-induced cell wall stiffening, in stomatal movement, and in programmed cell death (Angelini *et al.*, 2008). Although several studies have demonstrated a role for PAs in protection against abiotic stresses (Bouchereau *et al.*, 1999; Kasinathan and Wingler, 2004), little is known about how they act under conditions of biotic stress.

Plants are frequently exposed to a myriad of biotic and abiotic stresses that can act in succession or simultaneously. Being sessile organisms, plants have developed sophisticated acclimation and defence mechanisms to cope with different stress situations (Bray *et al.*, 2000; Boyko and Kovalchuk, 2011; Reddy *et al.*, 2011). These can be activated in the initial tissue exposed to stress as well as in systemic tissues that have not yet been exposed to the stress. Looking at the source of the stimulus, several kinds of induced resistance can be distinguished. If the induced resistance is achieved by treatment with a chemical or natural compound, this phenomenon is known as priming. Priming enables cells to respond to low levels of a stimulus in a more rapid and robust manner than is found in non-primed cells (Conrath, 2011). Thus, when plants are primed and subsequently challenged by pathogens or abiotic stresses, they show a faster and/or stronger activation of defence responses (Prime-A-Plant Group *et al.*, 2006). Recently, it has been demonstrated that hexanoic acid (Hx) induces resistance in *Solanum lycopersicum* and *Arabidopsis thaliana* plants against *Botrytis cinerea* (Vicedo *et al.*, 2009; Kravchuk *et al.*, 2011) and against *Pseudomonas syringae* pv tomato DC3000 (*Pst*) (Schalschi *et al.*, 2013) as well as in citrus plants against the fungus *Alternaria alternata* (Llorens *et al.*, 2013). The activation of defence or acclimation mechanisms in systemic non-challenged tissues is normally termed systemic acquired resistance (SAR)—if the local stimulus is induced by viruses, bacteria, or fungi—or systemic acquired acclimation (SAA)—if the initial stimulus is an abiotic stress situation—and serves an important role in preventing further infection or damage to the entire plant (Baxter *et al.*, 2014). Recent studies have demonstrated that the reactive oxygen species (ROS) wave functions as a general priming signal in plants, warning systemic tissues what is happening is a localized abiotic stress stimulus (Mittler *et al.*, 2011). Upon abiotic stress, SAA is mediated by temporal–spatial interactions of the ROS wave with hormone or amino acid signals activated in systemic tissues (Suzuki *et al.*, 2013). Specifically, recent reports demonstrated that acclimation-induced cross-tolerance in tomato plants is largely attributed to respiratory burst oxidase homologue (RBOH1)-dependent H_2_O_2_ production at the apoplast, which may subsequently activate MPK1/2 to induce stress responses (Zhou *et al.*, 2014). In recent years there has been evidence to suggest that plants with increased activation of response mechanisms by acclimation to abiotic stimuli can respond better to biotic stress, although little is known about the mechanisms underlying this type of induced resistance. Mild chronic stress situations can also boost plant stress resistance through induction of acclimation responses. Factors such as light, temperature, drought, mineral concentrations, and biotic infection are all capable of causing extensive damage to plants as well as inducing short- and long-term acclimation responses (Gordon *et al.*, 2013).

NH_4_^+^ is a fundamental substrate for amino acids, nucleic acids, alkaloids, and polysaccharides, as well as for secondary metabolites such as PAs (Bagh *et al.*, 2004), in all living organisms (von Wirén and Merrick, 2004). The downstream molecular events produced by NH_4_^+^ nutrition have been extensively studied and are related mainly to cell wall stability and biosynthesis, carbon metabolism and energy, primary N metabolism, phytohormones, and signalling molecules (Ariza *et al.*, 2013). However, NH_4_^+^ is toxic to cells when it is present at high concentrations in the soil or the nutrient solution because it causes the so-called ‘ammonium syndrome’. This may include leaf chlorosis, lower plant yield production and root/shoot ratio, lower cation content, acidification of the rhizosphere, and changes in several metabolite levels, such as amino acids or organic acids (Britto and Kronzucker, 2002; Bittsánszky *et al.*, 2015). Despite this, recent studies regarding the possible effect of NH_4_^+^ nutrition as an inducer indicate that NH_4_^+^ or one of its assimilation products (e.g. glutamine or glutamate) may serve as a stress signal and, in NH_4_^+^-grown plants, operate metabolic pathways that induce the accumulation of ROS (Misra and Gupta, 2006). Specifically, Fernández-Crespo *et al.* (2012) demonstrated that NH_4_^+^ nutrition confers protection against subsequent salt stress by reducing Cl^–^ uptake and decreasing its toxicity by priming accumulation of ABA and PAs, and by enhancing the basal content of H_2_O_2_ and proline in citrus plants. The authors concluded that NH_4_^+^ nutrition triggers mild chronic stress, which may account for the noted stress-induced morphogenetic responses (SIMRs) as part of a general acclimation strategy. The induction of the ‘acclimation stage’ leads to better adaptation to subsequent salt stress. Moreover, Fernández-Crespo *et al.* (2014) demonstrated that H_2_O_2_ and the manipulation of the antioxidant machinery act as intermediaries between mild stress induced by NH_4_^+^ nutrition and the development of the acclimation stage.

In this work, the effectiveness of NH_4_^+^ nutrition as an inducer of resistance against a biotic stress was tested, selecting for this purpose the pathogen *Pst*. Evidence is provided that NH_4_^+^ nutrition induces resistance against *Pst* in tomato plants, and assays were performed to determine the mode of action. It was concluded that NH_4_^+^ nutrition provokes mild toxicity in tomato plants, inducing H_2_O_2_ accumulation, which acts as a signal that can activate SAA and thus impart resistance to subsequent biotic stress. Moreover, the importance of Put and ABA downstream signalling pathways in NH_4_^+^-induced resistance (NH_4_^+^-IR) against *Pst* infection was demonstrated.

## Materials and methods

### Plant material, growth conditions, and nutrition treatments

Four-week-old tomato plants (*Solanum lycopersicum* Mill. cv. Ailsa Craig) were germinated in vermiculite in a growth chamber under the following environmental conditions: light/dark cycle of 16/8h, temperature of 24/18 °C, light intensity of 200 μmol m^–2^ s^–1^, and relative humidity of 60%. Seeds were irrigated twice a week with distilled water. Seedlings were irrigated for 3 weeks with Hoagland solution (Hoagland and Arnon, 1950) (control plants) or with Hoagland solution lacking N complemented with 20mM KNO_3_^–^ (NO_3_^–^ plants), or 2, 5, or 8mM NH_4_^+^ [(NH_4_)_2_SO_4_] (N-NH_4_^+^ plants). Then, K_2_SO_4_ and CaSO_4_ were added to compensate for the absence of K^+^ and Ca^2+^. The pH of the nutrient solution was adjusted to 6.0 with 1mM KOH.

Tomato genotypes used in the study were wild-type Ailsa Craig, Moneymaker, and Castlemart. The authors are grateful to Jonathan Jones (John Innes Centre, Norwich, Norfolk, UK) for seeds of the SA-deficient *NahG* tomato plant in the background Moneymaker, and to G. Howe (Michigan State University, East Lansing, MI, USA) for the JA pathway mutant *def1* in the background Castlemart. The ABA pathway mutant used was the ABA-deficient mutant *flacca* in the background Ailsa (LA3613), which was provided by the Tomato Genetics Resource Center (TGRC), University of California, Davis, CA, USA.

### Treatments with Put and PA biosynthesis inhibitors (DFMA and DFMO)

The chemical PA biosynthesis inhibitors difluoromethylarginine (DFMA) and difluoromethylornithine (DFMO), obtained from Dr Altabella, Centre for Research in Agricultural Genomics (CRAG), were dissolved in water and an inhibitor solution containing 2mM DFMA and 5mM DFMO was produced. Four treatments were performed, with the inhibitor solution was applied directly to each pot during the week prior to *Pst* inoculation. For Put treatment, 4-week-old plants were treated with 0.5mM Put or mock solution (water) using foliar sprays applied 48h before *Pst* infection. Tomato plants were maintained in the same culture conditions and inoculated as described above.

### *Pseudomonas syringae* and *Botrytis cinerea* bioassays

*Pst* was grown in King’s B (KB) medium (King *et al.*, 1954) at 28 °C. Rifampicin was added to KB medium at a concentration of 50mg ml^–1^. The coronatine-less strain of *Pst* (*CmaA*) (COR^–^) (Brooks *et al.*, 2005) was grown in KB medium with rifampicin (50mg ml^–1^) and kanamycin (25mg ml^–1^). For inoculation, *Pst* was grown in KB medium at 28 °C for 24h. Bacterial suspensions were adjusted to 5×10^5^ colony-forming units (cfu) ml^–1^ in sterile MgSO_4_ (10mM) containing 0.01% of the surfactant Silwet L-77 (Osi Specialties, Danbury, CT, USA), as described previously (Katagiri *et al.*, 2002). Pathogen inoculation was performed by dipping the third and fourth leaves into the bacterial suspension. The disease rate was scored at 72h post-inoculation (hpi) by determining the percentage of dark-brown spots on the leaf surface. For molecular and hormonal analyses, the samples were taken at 3, 24, and 48 hpi. At least three samples for colony counting and 20 samples for disease rate scoring were taken for each treatment over a 3 d period. Each experiment was independently conducted at least three times.

*Botrytis cinerea* CECT2100 was routinely cultured on potato dextrose agar at 24 °C. The *B. cinerea* spores were collected from 10- to 15-day-old cultures with sterile water containing 0.01% (v/v) Tween-20, which was then filtered, quantified with a haemacytometer, and adjusted at 1×10^6^ ml^–1^.

### Biomass, chlorophyll content, and photosynthetic rate (*A*_N_)

Four-week-old tomato plants (control and N-NH_4_^+^ plants) were collected and dried in an oven during 2 d at 65 ºC. Dried plant tissues were weighed, and the dry weight (DW) of 10 plants was obtained and expressed as biomass.

The chlorophyll level of the leaves of 4-week-old tomato plants was measured using a chlorophyll meter (SPDA; Minolta, Tokyo, Japan). Three measurements were taken per leaf on each side of the central vein, with 10 plants per treatment. The three SPAD readings taken on one leaf for each of the 10 plants per treatment were averaged to represent one observation. The results were obtained as SPAD values (arbitrary units). Although the equations used to convert the SPAD value into the chlorophyll concentration have been obtained for other species such as *Quercus serrata* (Hoshino, 1996), there is no such equation known for tomato plants to date.

For the net photosynthetic rate (*A*_N_) an LCpro+ portable infrared gas analyser (ADC BioScientific Ltd., Hoddesdon, UK) was utilized under ambient CO_2_ and humidity. Supplemental light was provided by a PAR lamp at 1000 μmol m^−2^ s^−1^ photon flux density and air flow was set at 150 μmol mol^−1^. After instrument stabilization, measurements were taken on three mature leaves (from an intermediate position on the stem) in each of the 10 replicate plants.

### H_2_O_2_ determination, microscopy analysis, and quantification

Samples of 10 leaves were collected for 3′,3-diaminobenzidine (DAB) staining at 3, 24. and 48 hpi. Leaves were cut and put immediately in 1mg ml^–1^ DAB at pH <3 for 24h in the dark and were subsequently destained in 96% ethanol and rehydrated in distilled water. DAB staining intensities were quantified in micrographies by the number of dark-brown DAB pixels in relation to the total pixels corresponding to plant material using the GIMP program (version 2.6.12).

H_2_O_2_ accumulation in leaves was quantified using the xylenol orange method (N.H. Kim *et al.*, 2013). Ten leaf discs (0.5cm^2^) were floated on 1ml of distilled water for 1h, centrifuged for 1min at 12 000 *g*, and 100 μl of supernatant was immediately added to 1ml of xylenol orange assay reagent. The mixture was incubated for 30min at room temperature. A standard curve for H_2_O_2_ was generated from measurements obtained from a serial dilution of 100nM to 100mM of H_2_O_2_. H_2_O_2_ was quantified by measuring the *A*_560_ using a spectrophotometer.

### Analysis of gene expression by quantitative real-time polymerase chain reaction (qRT-PCR)

Gene expression analysis by qRT-PCR was performed using RNA samples extracted from leaf tissue using the E.Z.N.A.^®^ Plant RNA Kit (www.omegabiotek.com), according to the manufacturer’s instructions. Tomato leaf tissue samples for RNA isolation were collected at 3 and 48 hpi. Leaf tissue from 10 plants each of the NH_4_^+^-treated and untreated plants was collected. A total of 1.5 μg of total RNA was digested using 1U of RNase-free DNase (Promega; http://www.promega.com) in 1 μl of DNase buffer and up to 10 μl of Milli-Q water, and was incubated for 30min at 37 ºC. After the incubation, 1 μl of RQ1 DNase stop buffer was added, and the solution was incubated again at 65 ºC for 10min to inactivate the DNase. Highly pure RNA was used for the reverse transcription reaction which was performed according to the instructions for the Omniscript reverse transcriptase kit (QIAGEN; http://www.qiagen.com/). The reaction mixture was incubated at 37 ºC for 60min. Forward and reverse primers (0.3 μM) were added to 12.5 μl of QuantiTect^™^ SYBR Green PCR reaction buffer (QIAGEN), as were 2 μl of cDNA and Milli-Q sterile water up to a total reaction volume of 25 μl. Quantitative PCR was carried out using the Smart Cycler II instrument (Cepheid; http://www.cepheid.com). A list of the primers used in the qRT-PCR is shown in Supplementary Table S1 available at *JXB* online. Levels of *EF1α* gene expression were used as an internal housekeeping control. Melting curve analysis was performed at the end of the PCR to confirm the purity of the amplified products.

The amplification efficiency for each primer pair was calculated using serial cDNA dilutions. Differences in cycle numbers during the linear amplification phase between samples from treated and untreated plants were used to determine differential gene expression. At least three independent experiments were performed to confirm the results. In each experiment, three biological replicates were used to generate means and determine the statistical significance.

### Stomatal aperture analysis

Tomato plants were maintained in the same culture conditions and treated as described above. The third and fourth leaves were inoculated with *Pst* collected from the plants at 1 and 3 hpi, and placed on glass slides with the adaxial epidermis in contact with dental resin. The dental resin mould was filled with nail polish to create a cast that was examined by light microscopy (Scalschi *et al.*, 2013). For stomatal aperture analysis, images of random regions were taken using a Leica IRB microscope equipped with a LeicaDC300F camera (Leica Microsystems CMS GmbH, Wetzlar, Germany). The stomatal aperture was analysed using the Eclipse-Net software of the Laboratory imaging program (http://www.laboratory-imaging.com). Approximately 50 stomata from each leaflet were measured.

### Chromatographic analysis

For hormonal analysis, fresh material was frozen in liquid N, ground, and freeze dried. Fresh tissue (0.5g) was immediately homogenized in 2.5ml of ultrapure water, and a mixture of internal standards ([^2^H_6_]ABA, [^2^H_4_]SA, dihydrojasmonic acid, and propylparaben) was added at 100ng ml^–1^ prior to extraction. After extraction, a 20 μl aliquot was injected directly into the high-performance liquid chromatography (HPLC) system. For PA analysis, fresh material was frozen in liquid N. Before extraction, according to the method described by Sánchez-López *et al.* (2009), a mixture of internal standards containing [^13^C_4_]putrescine and 1,7-diamineheptane was added. A 20 μl aliquot of this solution was directly injected into the HPLC system.

Analyses of hormone and PA samples were carried out using a Waters Alliance 2690 HPLC system (Milford, MA, USA) with a nucleosil ODS reversed-phase column (100 mm×2mm, i.d. 5 μm; Scharlab, Barcelona, Spain; http://www.scharlab.com). The chromatographic system was interfaced to a Quatro LC (quadrupole–hexapole–quadrupole) mass spectrometer (Micromass; http://www.micromass.co.uk). The MASSLYNX NT software version 4.1 (Micromass) was used to process the quantitative data from calibration standards and plant samples.

### Statistical analysis

Statistical analysis was carried out using a one-way analysis of variance in the Statgraphics-plus software of Windows V.5 (Statistical Graphics Corp., Rockville, MD, USA). The means are expressed with standard errors and compared using a Fisher’s least-significant difference test at the 95% confidence interval. All of the experiments were repeated at least three times.

## Results

### NH_4_^+^ nutrition enhances tomato resistance against *Pst* infection

**Fig. 1.**
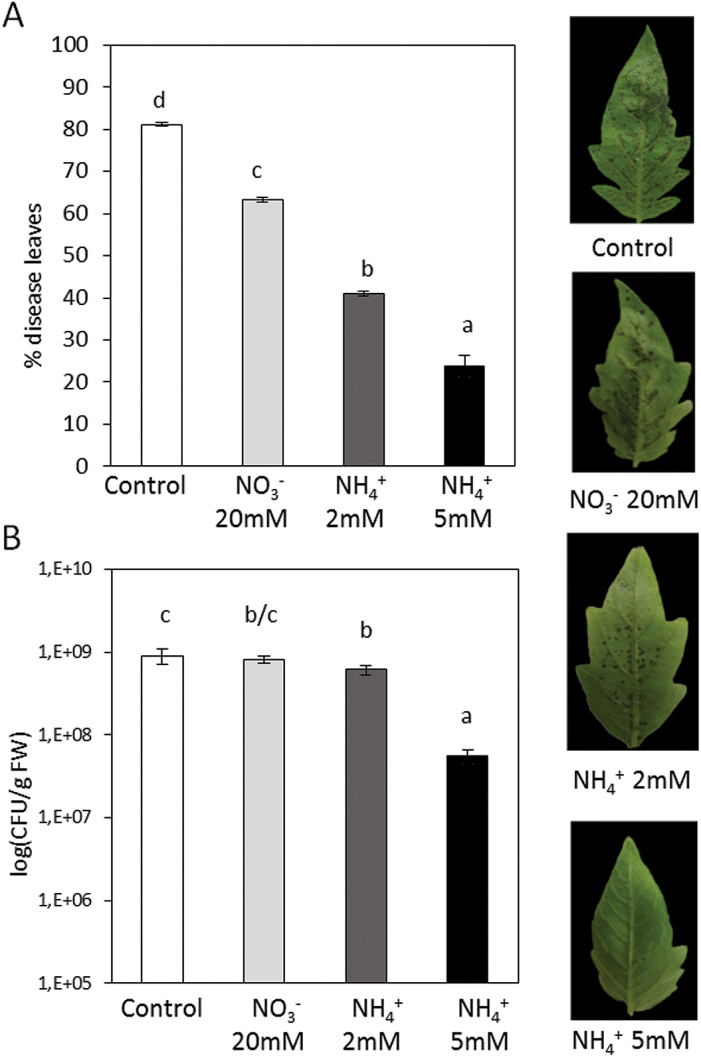
Effect of different N treatments on the resistance of tomato plants to *Pst*. Four-week-old tomato plants grown with different NO_3_^–^ and NH_4_^+^ concentrations were inoculated by dipping them in a bacterial suspension of *Pst* at 5×10^5^ cfu ml^−1^. At 72 hpi, the disease rating was scored by measuring the percentage of infected leaves in relation to the total number of analysed leaves (A) and by recounting of bacterial populations by plating in agar–KB medium (B). The photograph shows a representative picture of disease symptoms in the different N treatments. Data show the average of three independent experiments of a pool of 10 plants per experiment ±SE. Letters indicate statistically significant differences (*P*<0.05; least-significant difference test). (This figure is available in colour at *JXB* online.)

To investigate the role of NH_4_^+^ nutrition in induced resistance against *Pst*, 4-week-old tomato plants of *S. lycopersicum* were germinated in vermiculite under growth chamber-controlled conditions. Seeds were irrigated twice weekly with Hoagland complete solution (control plants) or with Hoagland solution lacking N completed with different NO_3_^–^ and NH_4_^+^ concentrations [20mM KNO_3_^–^, N-NO_3_^–^ plants; and 2, 5, and 8mM (NH_4_)_2_SO_4_, N-NH_4_^+^ plants]. All plant groups were inoculated with *Pst* by dipping leaves into the bacterial suspension. Statistically significant reductions in disease symptoms (Fig. 1A) and in the size of the bacterial population (Fig. 1B) were found at 72 hpi under all N nutrition conditions. This reduction was more pronounced in plants grown with 5mM NH_4_^+^, demonstrating that the inducer effect of NH_4_^+^ nutrition is concentration dependent. This direct correlation between NH_4_^+^ concentration and *Pst* resistance is lost at high concentrations of this ion since clear toxicity symptoms were observed in tomato plants grown with 8mM NH_4_^+^ (data not shown). To gain further insight into the biochemical and molecular mechanisms related to NH_4_^+^-mediated resistance to *Pst*, the 5mM nutrition condition was selected for use in this study. This concentration was also tested against a necrotrophic pathogen, *B. cinerea*, and a similar effect was observed (Supplementary Fig. S2 at *JXB* online).

### NH_4_^+^ nutrition induces mild toxicity, the first step of the acclimation stage

NH_4_^+^ is a paradoxical nutrient ion because, despite being a major N source for many metabolic reactions, it can provoke toxic symptoms in sensitive species. In this study, the influence of NH_4_^+^ nutrition on plant growth, chlorophyll content, and photosynthetic rate (*A*_N_) (Table 1) was analysed. As expected, plants grown with NH_4_^+^ as the sole source of N showed a 27% reduction in their growth, when compared with control plants. Moreover, it was observed that NH_4_^+^ nutrition provoked a significant increase in chlorophyll content and induced *A*_N_ inhibition in tomato plants.

**Table 1.** Effect of NH_4_^+^ nutrition on growth, chlorophyll content, and photosynthetic rate (A_N_)

Treatments	Biomass (g DW)	Cholorophyll content (SPAD units)	*A*_N_ (μmol m^−2^ s^−1^)
Control	0.480±0.117 b	37.573±0.578 b	21.565±0.841 b
NH_4_^+^	0.345±0.031 a	43.300±0.716 a	13.543±0.663 a

### Put accumulation is required for NH_4_^+^-IR against *Pst*

**Fig. 2.**
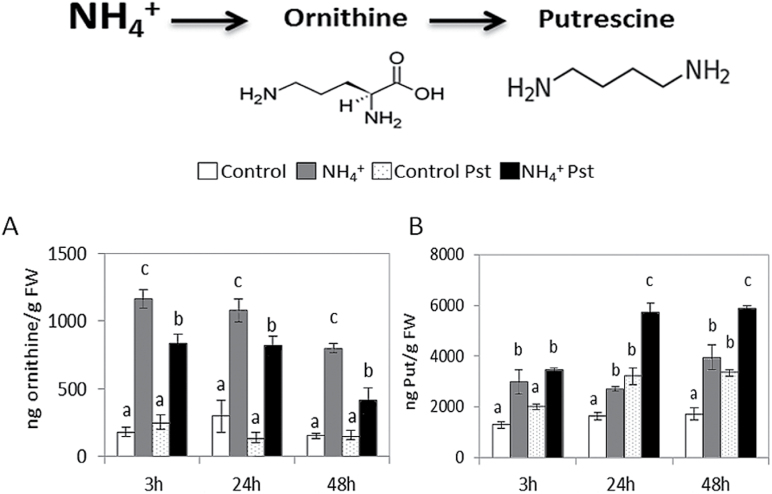
PA content in control and N-NH_4_^+^ tomato plants following *Pst* infection. Four-week-old tomato plants were grown under control conditions or with 5mM NH_4_^+^, and inoculated by dipping them in a bacterial suspension of *Pst* at 5×10^5^ cfu ml^−1^. Leaves were collected at various time points, and ornithine (A) and Put (B) levels were determined in freeze-dried material by HPLC–MS. Data show the average of three independent experiments of a pool of 10 plants per experiment ±SE. Letters indicate statistically significant differences (*P*<0.05; least-significant difference test).

To determine whether NH_4_^+^ nutrition affects the PA content, ornithine, Put, Spm, and Spd levels were determined at 3, 24, and 48 hpi in control and N-NH_4_^+^ plants (Fig. 2; Supplementary Fig. S1 at *JXB* online). N-NH_4_^+^ plants displayed higher basal levels of ornithine and Put content when compared with control plants. However, upon infection, no changes in ornithine content were observed in control plants, while infected N-NH_4_^+^ plants showed a marked decrease in ornithine content, which was apparently transformed into Put. Regarding Put accumulation, although both control and N-NH_4_^+^ plants displayed an increase in Put content at 24 and 48 hpi, the increase was higher in infected N-NH_4_^+^ plants (Fig. 2). No changes were observed in the Spm and Spd content under any experimental condition (Supplementary Fig. S1). Collectively, these results suggest the involvement of PA metabolism, specifically of Put, in NH_4_^+^-IR.

**Fig. 3.**
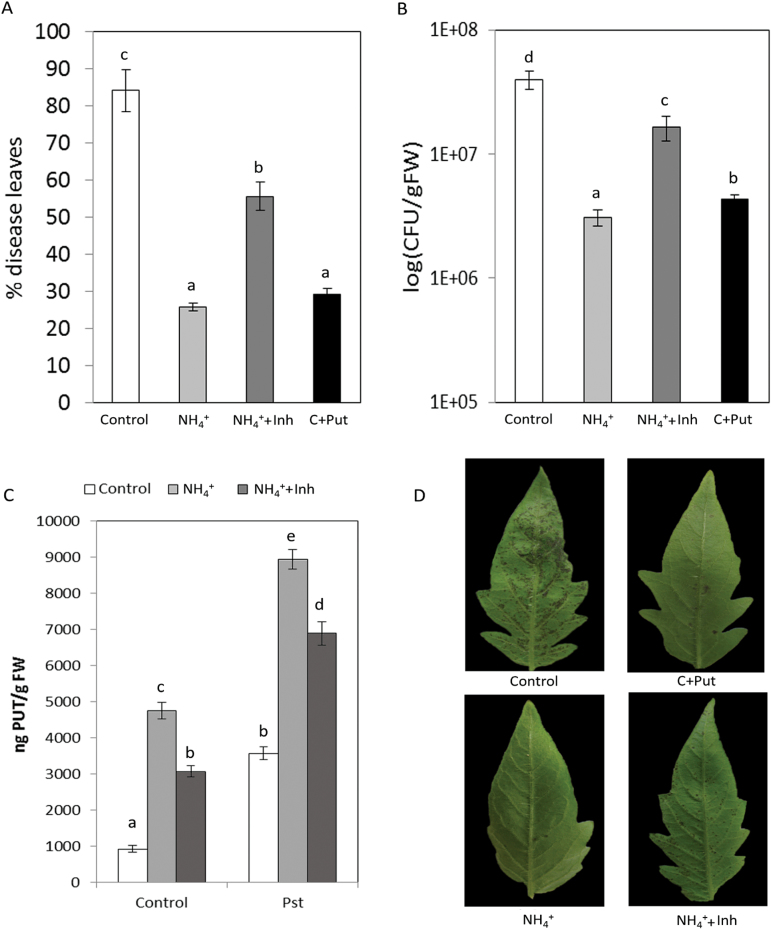
Influence of Put accumulation on NH_4_^+^-IR against *Pst*. Plants were grown as described in Fig. 2 and, 1 week prior to inoculation, N-NH_4_^+^ plants were treated with inhibitor solution (containing DFMO and DFMA; NH_4_^+^+Inh) and control plants were treated with 0.5mM Put 48h before infection (C+Put), and inoculated by dipping them in a bacterial suspension of *Pst* at 5×10^5^ cfu ml^−1^. The disease rating was scored by measuring the percentage of infected leaves in relation to the total number of analysed leaves (A) and by recounting of bacterial populations by plating in agar–KB medium (B) at 72 hpi. The Put level in control, NH_4_^+^, and NH_4_^+^+Inh plants was analysed at 48 hpi (C). The photograph shows a representative picture of disease symptoms in control, NH_4_^+^, NH_4_^+^+Inh, and C+Put tomato leaves at 72 hpi (D). Data show the average of three independent experiments of a pool of 10 plants per experiment ±SE. Letters indicate statistically significant differences (*P*<0.05; least-significant difference test). (This figure is available in colour at *JXB* online.)

To assess the importance of Put accumulation, DFMA and DFMO were used as irreversible inhibitors of ADC and ODC enzymes, respectively (Fallon and Phillips, 1988), with the purpose of reducing cellular Put accumulation induced by NH_4_^+^ nutrition (Fig. 3). As expected, N-NH_4_^+^ plants treated with inhibitor solution displayed a reduction of 35.3% in Put content in the absence of *Pst* (Fig. 3C). Surprisingly, N-NH_4_^+^ plants treated with the inhibitor solution were more susceptible to *Pst* when compared with untreated N-NH_4_^+^ plants (Fig 3A, B, D). This shows that reduced Put accumulation reverts the resistance phenotype induced by NH_4_^+^ nutrition, revealing an important role for Put accumulation. To confirm the effect of Put on *Pst* infection, control plants were treated with 0.5mM Put by foliar spray 48h before infection. It was observed that, in control plants, Put induced statistically significant reductions in disease symptoms (Fig. 3A) and bacterial population size (Fig. 3B). These experiments demonstrate that Put accumulation is a crucial event in the resistance of tomato plants to *Pst*.

### Oxidative burst induced by NH_4_^+^ nutrition enhances the resistance against *Pst*

**Fig. 4.**
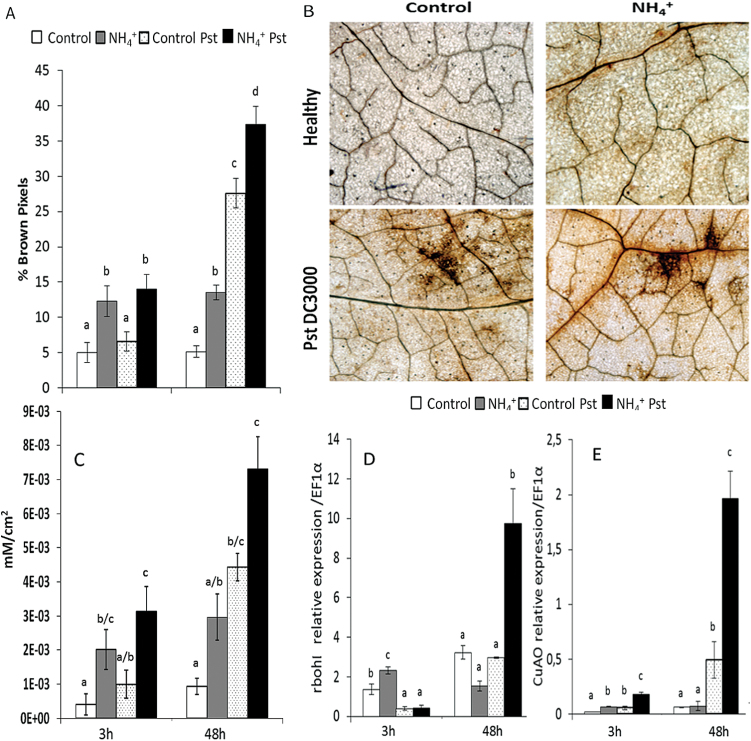
Effect of NH_4_^+^ nutrition on the oxidative burst upon *Pst* infection. Plants were grown and inoculated as described in Fig. 2. H_2_O_2_ accumulation was visualized by DAB staining. Quantification was performed by determining the number of brown pixels on digital photographs of leaves at 3 and 48 hpi (A). Representative photographs were taken of H_2_O_2_ accumulation in control and N-NH_4_^+^ plants in the absence of pathogens, as well as 48 hpi (B). Data show average values ±SE (*n*=20) of the relative number of brown or yellow pixels per photograph. H_2_O_2_ concentrations were quantified by xylenol orange analysis (C). Total RNA was isolated from leaves at 3 and 48 hpi and was converted into cDNA and subjected to a qRT-PCR analysis. The relative level of (D) *rboh1* and (E) *CuAO* was analysed in the control and N-NH_4_^+^ plants. The results were normalized to the *EF1α* gene expression measured in the same samples. Letters indicate significant differences between treatments at each time point (*P*<0.05; least-significant difference test). (This figure is available in colour at *JXB* online.)

Early accumulation of ROS is one of the first biochemical responses of the plant to pathogen attack. To clarify how NH_4_^+^ nutrition affects cellular oxidative burst, and to determine its relationship to NH_4_^+^-IR against *Pst*, H_2_O_2_ accumulation was evaluated by means of DAB staining (Fig. 4). H_2_O_2_ accumulation was examined at 3 and 48 hpi in control and N-NH_4_^+^ plants by digital quantification of DAB intensity (Fig 4A–C). This analysis revealed that N-NH_4_^+^ plants showed higher basal levels of H_2_O_2_ accumulation than control plants. Upon infection with *Pst*, no changes in H_2_O_2_ accumulation were observed at 3 hpi. However, at 48 hpi, *Pst* induced strong oxidative bursts in control and N-NH_4_^+^ plants, although the highest levels of H_2_O_2_ accumulation occurred in the latter. These results indicate that cellular oxidative stress could have an important role in NH_4_^+^-IR against *Pst*. Thus, respiratory burst oxidase *rboh1* and *CuAO* gene expression was analysed at 3 and 48 hpi by qRT-PCR (Fig 4D, E). In N-NH_4_^+^ plants, higher *rboh1* mRNA accumulation was observed at 3h in the absence of *Pst*. At 48 hpi, N-NH_4_^+^ plants displayed strong *rboh1* induction when compared with control plants. Similar results to those obtained for *rboh1* were observed in the analysis of *CuAO.*

### NH_4_^+^ nutrition induces changes in the hormonal profile of tomato plants

**Fig. 5.**
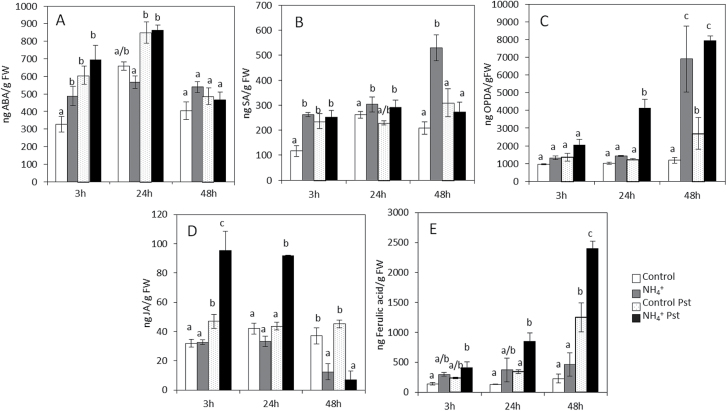
Hormonal profile in control and the N-NH_4_^+^ tomato plants upon *Pst* infection. Plants were grown and inoculated as described in Fig. 2. Leaves were collected at different time points, and ABA (A), SA (B), OPDA (C), JA (D), and ferulic acid (E) levels were determined by HPLC–MS. The concentration of the hormones was determined in all samples by normalizing the chromatographic area for each compound with the fresh weight of the corresponding sample. Data show the average of three independent experiments of a pool of 20 plants per experiment ±SE. Letters indicate statistically significant differences at each time point (*P*<0.05; least-significant difference test).

To determine whether the main signalling pathways are involved in NH_4_^+^-IR, the levels of hormones and ferulic acid were analysed simultaneously in control and N-NH_4_^+^ plants at 3, 24, and 48 hpi (Fig. 5). N-NH_4_^+^ plants displayed higher basal ABA and SA levels compared with control plants. On infection with *Pst*, no changes in ABA and SA content were observed in treated plants during the experiment. In the oxylipin pathway, a significant increase was observed in the 12-oxophytodienoic acid (OPDA) content of the infected N-NH_4_^+^ plants at 24 and 48 hpi compared with the control plants. A fast increase in the JA level in response to *Pst* at 3 hpi was found in the N-NH_4_^+^ condition, whereas no changes were found in control and infected plants. These results showed that NH_4_^+^ nutrition induces fast activation of oxylipin pathways in tomato plants, which leads to significant OPDA accumulation. Despite that, an abolished production of JA-Ile levels was observed in these plants (data not shown). Moreover, an increase in the ferulic acid content was observed at 48 hpi, which was higher in the infected N-NH_4_^+^ plants.

### NH_4_^+^-IR against *Pst* involves ABA signalling pathways

**Fig. 6.**
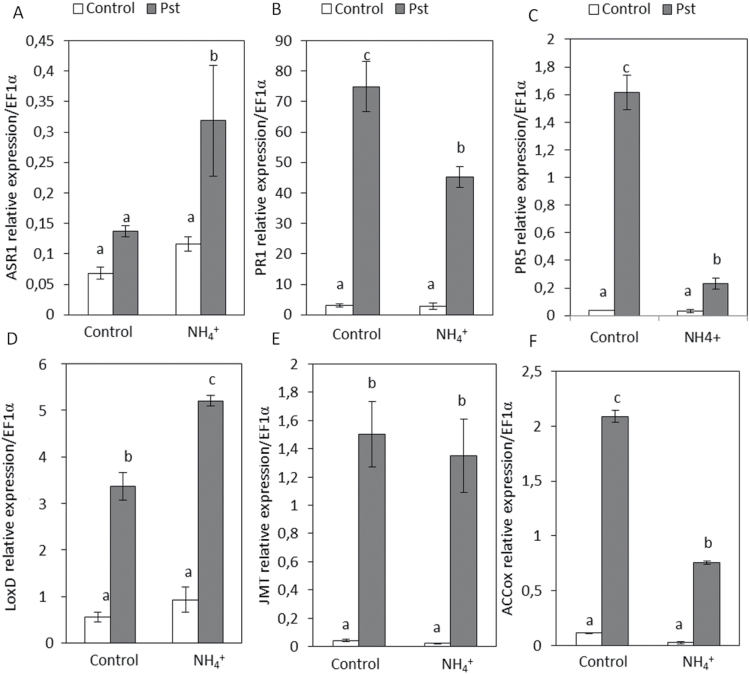
Gene expression profile of plant defence pathways in control and N-NH_4_^+^ tomato plants upon *Pst* infection. Plants were grown and inoculated as described in Fig. 2. The expression of genes representing key components of ABA (*Asr1*) (A), SA (*PR1* and *PR5*) (B and C), JA (*LoxD* and *JMT*) (D and E), and ethylene (*ACCOx*) (F) signalling pathways were analysed in cDNA from leaves of control and N-NH_4_^+^ plants at 48 hpi. The results were normalized to the *EF1α* gene expression measured in the same samples. Data show the average of three independent experiments of a pool of 10 plants per experiment ±SE. Letters indicate statistically significant differences (*P*<0.05; least-significant difference test).

To confirm the changes in hormonal balance produced by NH_4_^+^ nutrition, the expression patterns of marker genes for ABA (*Asr1*), SA (*PR1* and *PR5*), JA (*LoxD, JMT*), and ethylene (*ACCOx*) signalling pathways were analysed in the control and N-NH_4_^+^ plants at 48 hpi (Fig. 6). NH_4_^+^ nutrition induces *Asr1* mRNA accumulation at 48 hpi, although an increase in ABA levels was not observed at this time point. However, in the absence of infection, N-NH_4_^+^ plants displayed higher basal levels of ABA at early time points. This initial ABA accumulation and the strong induction of *Asr1* suggest that the ABA pathway has an important role in NH_4_^+^-IR. Regarding SA pathways, although N-NH_4_^+^ plants showed higher basal SA levels compared with control plants, upon infection no significant increase in SA content was observed. However, control and N-NH_4_^+^ plants displayed larger increases in *PR1* and *PR5* mRNA accumulation in response to *Pst* infection, being more pronounced in control infected plants. The expression patterns of marker genes related to oxylipin pathways revealed that *Pst* infection induces *LoxD* expression at 48 hpi, but its expression was higher in N-NH_4_^+^ plants. Regarding *JMT* expression, both control and N-NH_4_^+^ plants displayed an increase in mRNA accumulation at 48 hpi without significant changes between treatments. Moreover, the analyses were extended to the *ACCox* marker gene related to the ethylene pathway and it was observed that, in control and N-NH_4_^+^ plants, *Pst* infection enhances *ACCox* mRNA accumulation, although this was higher in control plants. Based on these results, it can be concluded that the higher induction of *Asr1* and *LoxD* genes points to the fact that NH_4_^+^-IR might be due to the activation of ABA and JA defence signalling pathways.

**Fig. 7.**
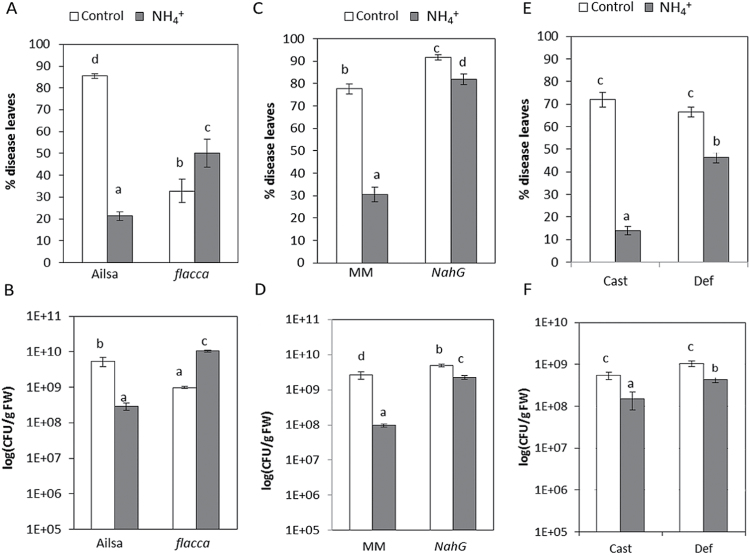
Influence of ABA, SA, and JA signalling pathways on NH_4_^+^-IR against *Pst*. Four-week-old tomato mutants impaired in these signalling pathways were grown under control and NH_4_^+^ treatments and inoculated by dipping in a bacterial suspension of *Pst*. The disease rating was scored for wild-type tomato plants of Ailsa Craig (Ailsa), Moneymaker (MM), and Castlemart (Cast) and their respective ABA-impaired mutant *flacca* (A, B), SA-impaired mutant *NahG* (C, D), and JA-impaired mutant *def1* (E, F) at 72 hpi. Data show the average of three independent experiments of a pool of 10 plants per experiment ±SE. Letters indicate statistically significant differences (*P*<0.05; least-significant difference test).

To gain further insight into the mechanisms behind NH_4_^+^-IR against *Pst*, tomato mutants impaired in the SA, ABA, or JA pathways were analysed (Fig. 7). NH_4_^+^ nutrition did not protect ABA-deficient mutant *flacca* plants against *Pst* (Fig, 7A, 7B). N-NH_4_^+^ *flacca* plants displayed significant increases in disease symptoms, as well as in the size of the bacterial population, compared with control *flacca* plants, indicating a requirement for this hormonal pathway in NH_4_^+^-IR. Transgenic *NahG* plants displayed a basal susceptibility to *Pst* due to their reduced SA levels. *NahG* plants displayed intact NH_4_^+^-IR, which supports the theory that SA does not play an important role in NH_4_^+^-IR (Fig. 7C, D). The mutant *def1*, which is deficient in JA biosynthesis and acts downstream of OPDA formation, showed intact NH_4_^+^-IR (Fig. 7E, F). This result supports the possible implication of oxylipin molecules upstream of OPDA in NH_4_^+^-IR.

### NH_4_^+^ nutrition induces basal stomatal closure in tomato plants

**Fig. 8.**
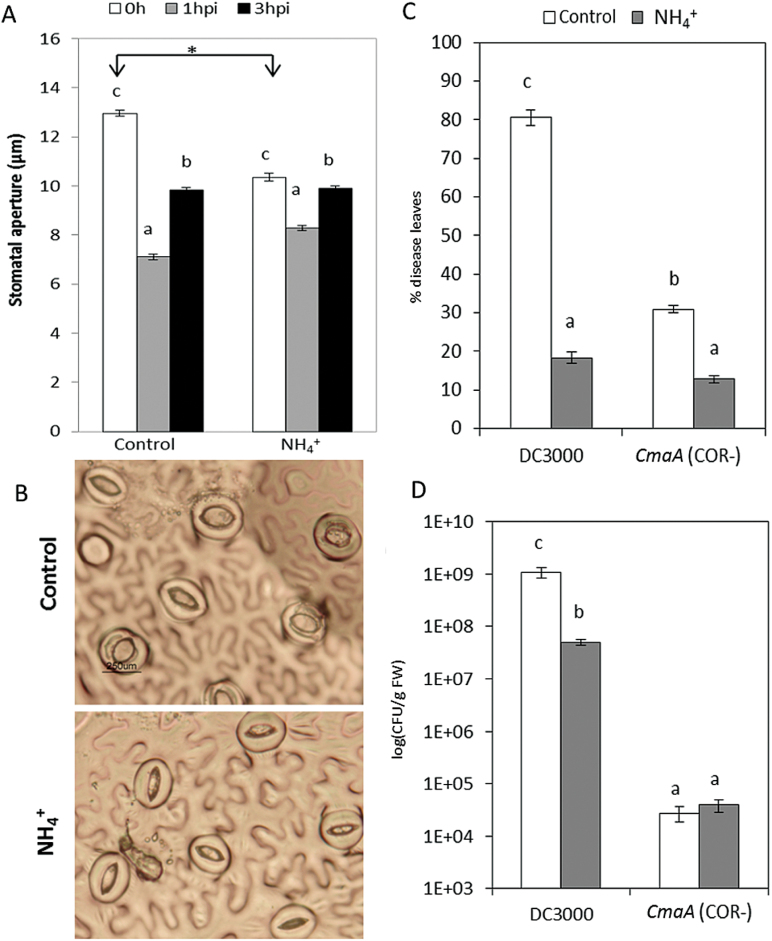
NH_4_^+^ treatment induces basal stomatal closure, and NH_4_^+^-IR against *Pst* is independent of the COR toxin effect. Tomato plants were grown, treated, and inoculated as described in Fig. 2. Stomatal apertures were analysed ‘*in situ*’ in leaflets of control and N-NH_4_^+^ plants at 0, 1, and 3 hpi (A). Results are means ± SE (*n*>50 stomata). Representative photographs of basal stomatal closure induced by NH_4_^+^ treatment (B) were taken. Tomato plants were infected by dipping them in a bacterial suspension of *Pst* and the coronatine-less strain of *Pst* (*CmaA* COR^–^). The disease rating was scored by measuring the percentage of infected leaves (C) and by recounting of bacterial populations (D) at 72 hpi. Data show the average of three independent experiments of a pool of 10 plants per experiment ±SE. Letters indicate statistically significant differences (*P*<0.05; least-significant difference test). (This figure is available in colour at *JXB* online.)

A cellular response induced upon PAMP recognition is the closure of stomata within a few hours to restrict pathogen spread to other plant tissues. Therefore, in order to invade the apoplast and cause disease, *Pst* produces the bacterial phytotoxin COR to impede stomatal closure and/or trigger stomatal reopening. To study the possible effect of NH_4_^+^ on this process, stomatal aperture was measured at 0, 1, and 3 hpi in control and N-NH_4_^+^ plants (Fig. 8). In both treatments, it was observed that plants closed their stomata to restrict pathogen entry into the apoplast at 1 hpi. Moreover, at 3 hpi, both control and N-NH_4_^+^ plants showed stomatal reopening, probably induced by COR, without significant changes between treatments (Fig. 8A). Curiously, N-NH_4_^+^ plants displayed basal stomatal closure when compared with control plants (Fig. 8B) which might contribute to the NH_4_^+^-IR against *Pst*. Thus, the relevance of the virulence factor COR, a JA-Ile mimic, on NH_4_^+^-IR was further tested. Control and N-NH_4_^+^ plants were infected with the *Pst* strain *cmaA*, which lacks COR (COR^–^). A reduction in bacterial growth was observed in plants infected with the mutant strain. Under NH_4_^+^ nutrition, a statistically significant reduction in disease symptoms was observed when compared with control plants (Fig. 8C). Although no significant changes were found in the size of the bacterial population (Fig. 8D), the reduction in disease symptoms and the normal reopening at 3 hpi in N-NH_4_^+^ plants revealed that this nutrition does not directly interfere with COR action.

## Discussion

The role of NH_4_^+^ as an essential macronutrient and signalling molecule has been extensively studied (Castaings *et al.*, 2011), but its impact on plant defence responses is still unclear. In this study, the complex relationship between SAA mechanisms and the NH_4_^+^-IR against *Pst* was clarified by observing that changes in the N nutrition status, specifically NH_4_^+^ applied as the sole N source, resulted in an increased resistance against *Pst* in tomato plants. NH_4_^+^ nutrition protects tomato plants in a concentration-dependent manner, and this resistance is especially important for plant growth under conditions of 5mM NH_4_^+^. Extensive studies investigating the underlying mechanisms of NH_4_^+^ toxicity have been reported in plants, but how plants acclimated to high levels of NH_4_^+^ are able to induce the mechanisms of resistance against subsequent stress situations more efficiently is poorly understood. Plants accumulate PAs to compensate for the lack of some cations other than NH_4_^+^, besides serving as a sink for excess NH_4_^+^ to reduce toxicity (Gerendás *et al.*, 1997). Although long known for their implication in abiotic stress responses, there has been little investigation of defence responses of PAs during pathogen infection. In this work, it was observed that N-NH_4_^+^ plants displayed a higher basal content of ornithine and Put. Upon infection, N-NH_4_^+^ plants showed a marked decrease in ornithine content, which was apparently transformed into Put. Interestingly, N-NH_4_^+^ tomato plants displayed more susceptibility to *Pst* when plants were treated with the inhibitors DFMA and DFMO, and Put content was reduced. Moreover, Put treatment induces resistance against this pathogen in control tomato plants, revealing the importance of PAs, specifically Put, in the resistance of tomato plants to a biotic stress. According to these findings, it can be confirmed that signalling derived from changes to the ornithine pool and its conversion in Put, leading to high Put accumulation, are directly linked to NH_4_^+^ nutrition and are key events in NH_4_^+^-IR against *Pst*. S.H. Kim *et al.* (2013) observed that an *adc2* knock-out mutant displayed reduced Put content, reduced expression of *PR1*, and enhanced susceptibility against *Pst*. Disease susceptibility of the *adc2* mutant was recovered by the addition of exogenous Put, revealing its direct impact on resistance against this pathogen. Another common response to the toxic effect of NH_4_^+^ nutrition is ROS accumulation and the modification of the redox cell state (Patterson *et al.*, 2010). Recent studies identified plastid retrograde signalling-derived responses as key factors in plants against NH_4_^+^ stress (Li *et al.*, 2012, 2013). These authors propose that, under NH_4_^+^ stress, the chloroplast receives the stress signal (mediated by ROS) and activates retrograde signalling pathways, recruiting downstream ABA signalling to regulate the expression of NH_4_^+^-responsive genes in the nucleus and prevent NH_4_^+^ toxicity. In this study, the mild toxic effect of NH_4_^+^ nutrition on tomato plants was confirmed since growth retardation, increased chlorophyll content, reduced photosynthetic rate, and basal H_2_O_2_ accumulation were observed in N-NH_4_^+^ plants. The plastid retrograde signalling induced by H_2_O_2_ accumulation is probably related to the expression of nuclear genes to prevent NH_4_^+^ toxicity. The activation of this defensive pathway might induce the establishment of SAA in leaves of tomato plants, allowing them to better withstand a subsequent *Pst* infection. This hypothesis is in accordance with previous studies, in which it was demonstrated that NH_4_^+^ nutrition in citrus plants triggers mild chronic stress, induces H_2_O_2_ accumulation, and acts as a signal, which primes plant defence responses by stress imprinting and confers protection against subsequent salt stress (Fernández-Crespo *et al.*, 2012, 2014). Although little is known about how prior exposure of plants to abiotic stress improves their capacity to respond effectively to biotic stress, recent studies suggest that ROS are closely associated with this adaptive process. For example, high light exposure induced ROS accumulation, and this signal is required for the SAA of plants, enhancing tolerance of plants to *Pst*, indicating that there is cross-talk between abiotic stress acclimation and pathogen responses (Karpinski *et al.*, 2013). For this reason, it was concluded that H_2_O_2_ accumulation derived from mild chronic NH_4_^+^ stress acts as a signal and primes plant defence responses, probably mediated by the activation of downstream plastid retrograde signalling. The signalling derived from the chloroplast could be related to the establishment of SAA in tomato plants, enhancing the resistance against subsequent *Pst* infection.

To investigate further the role of ROS in the SAA induced by NH_4_^+^ nutrition, *rboh1* and *CuAO* gene expression as H_2_O_2_ producers was analysed. As expected, NH_4_^+^ nutrition induced mRNA accumulation of both genes in tomato plants. RBOHs are required for the initiation and self-propagation of systemic signals by H_2_O_2_ accumulation to generate a ‘ROS wave’ (Mittler *et al.*, 2011). Suzuki *et al.* (2013) demonstrated that SAA of plants to heat stress was correlated with activation of the ROS wave and the transient accumulation of ABA in systemic tissues, and these responses were suppressed in a mutant lacking RBOHD. According to these findings, the higher basal induction of *rboh1*, accompanied by the higher basal H_2_O_2_ accumulation induced by NH_4_^+^ nutrition, might be the key event in the acclimation stage induced in tomato plants. As for CuAO, it is commonly accepted that H_2_O_2_ produced as a catabolite by CuAO action is involved in ABA-induced stomatal closure in *Vicia faba* (An *et al.*, 2008). Therefore, H_2_O_2_ produced by CuAO and RBOH1 could have a dual role; first it may play an important role in ABA-mediated basal stomatal closure, which was found in N-NH_4_^+^ tomato plants, and, secondly, the H_2_O_2_ wave could be used as an amplifier for signalling related to SAA mechanisms. N-NH_4_^+^ plants displayed a strong oxidative burst, probably mediated by the highest induction of the *rboh1* and *CuAO* gene. This response which leads to limited pathogen spread might play a key role in NH_4_^+^-IR against *Pst*.

To understand the effect of NH_4_^+^ nutrition on defence, signalling pathways were analysed. The hormonal profile revealed that NH_4_^+^ nutrition induces higher basal levels of SA in tomato plants, but upon *Pst* infection no significant changes in SA content between treatments were found. *PR1* and *PR5* gene expression is less induced in the infected NH_4_^+^ plants, and *NahG* plants displayed intact NH_4_^+^-IR. This evidence supports the hypothesis that SA signalling pathways are not the main pathways required for NH_4_^+^-IR against *Pst*. Regarding oxylipin pathways, a faster and stronger accumulation of JA in N-NH_4_^+^ plants at 3 hpi was observed. However, throughout the infection, decreased JA levels occurred accompanied by a higher increase in OPDA content and a strong induction of *LoxD* in N-NH_4_^+^ plants. The role of JA and OPDA in the NH_4_^+^-IR was tested with JA-deficient *def1* plants impaired in JA as well as OPDA accumulation, and an intact resistance against *Pst* was shown, revealing the possible implication of oxylipins upstream of OPDA in the NH_4_^+^-IR against *Pst*. Regarding the phenylpropanoid pathway, infected NH_4_^+^ plants displayed higher accumulation of ferulic acid, which points to the implication of this pathway in NH_4_^+^-IR. It is probable that shikimate derived from tyrosine competes with SA accumulation synthesized via phenylalanine ammonia lyase, and, thus, significant changes in free SA as well as reduced induction of *PR1* and *PR5* in N-NH_4_^+^ plants were not observed.

As expected, N-NH_4_^+^ plants displayed higher basal ABA accumulation and, on infection, NH_4_^+^ nutrition primed the induction of *Asr1*. To clarify the role of ABA-dependent signalling pathways, the effect of NH_4_^+^ nutrition on the responses of ABA-deficient *flacca* mutants was analysed. It was demonstrated that *flacca* NH_4_^+^-treated plants were more susceptible to *Pst*, and displayed impaired NH_4_^+^-IR expression. The fact that NH_4_^+^-IR was absent in *flacca* supports the idea that an intact ABA signalling pathway is required, at least in part, in NH_4_^+^-IR against *Pst.* ABA has also emerged as a complex modulator of plant defence responses, since it can function as a positive or negative regulator, depending on the plant–pathogen interaction analysed (Ton *et al.*, 2009). Specifically, ABA was found to be a key regulator of the pathogen-mediated stomatal closure (Melotto *et al.*, 2006). One mechanism of *Pst* pathogenesis is to produce the effector COR necessary for stomatal reopening in the process of bacterial infection (Melotto *et al.*, 2006). To confirm the role of NH_4_^+^ nutrition in the stomatal movement on *Pst* infection, the stomatal aperture was analysed in treated and untreated plants at 0, 1, and 3 hpi. As expected, it was observed that bacteria induced closure of stomata at 1 hpi, and stomata reopened in a COR-dependent manner at 3 hpi in control and N-NH_4_^+^ plants (Tsai *et al.*, 2011). However, N-NH_4_^+^ plants displayed more closed stomata than control plants, probably due to the higher basal ABA accumulation. These findings reveal that NH_4_^+^ does not have a direct effect on stomatal movement induced by COR, but the basal stomatal closure observed in NH_4_^+^ plants was sufficient to reduce the entry of bacterial into the mesophyll and reduce the disease symptoms. In addition to its role in stomatal movement, ABA and its role in PA homeostasis has been widely studied. Toumi *et al.* (2010) showed that ABA induced PA accumulation and secretion into the apoplast, where they were oxidized by CuAOs producing H_2_O_2_. Based on these findings, it seems clear that activation of ABA-dependent signalling pathways mediated by NH_4_^+^ nutrition may activate PA biosynthesis and catabolism. For this reason, it is concluded that ABA could act as a positive regulator of NH_4_^+^-IR since it was able to strengthen Put biosynthesis, which has a direct effect on the resistance to *Pst*, and induced PA catabolism by enhancing CuAO activity, causing H_2_O_2_ accumulation and therefore provoking basal stomatal closure.

**Fig. 9.**
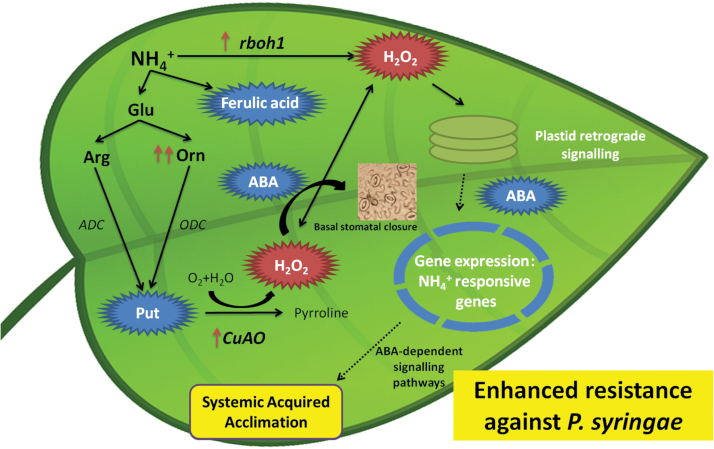
Model of achievement of SAA induced by NH_4_^+^ nutrition. Tomato plants grown in NH_4_^+^ as a sole N source develop different responses to relieve the mild toxicity effect. These responses play an important role in achieving SAA and in the resistance against *Pst*. A common response against NH_4_^+^ nutrition is the accumulation of PAs to compensate for the lack of some cations other than NH_4_^+^, besides serving as a sink for excess NH_4_^+^ to reduce the toxicity. Here, basal metabolic changes induced by NH_4_^+^ nutrition were observed, specifically Put, ABA, and ferulic acid accumulation. Another response against the mild toxic effect of NH_4_^+^ nutrition is ROS accumulation and, consequently, the modification of the redox cell state. This modification occurs by the basal induction of *CuAO* and *rboh1* genes observed in N-NH_4_^+^ plants. Moreover, it was found that basal stomatal closure is probably produced by H_2_O_2_ derived from enhanced *CuAO* activity, induced by ABA-mediated NH_4_^+^ responses. The chloroplast receives the stress signal (mediated by ROS) and activates retrograde signalling pathways, recruiting downstream ABA signalling to regulate the expression of NH_4_^+^-responsive genes in the nucleus and prevent NH_4_^+^ toxicity. The activation of this defensive pathway might induce the establishment of SAA in tomato plant leaves, allowing them to better withstand a subsequent *Pst*. (This figure is available in colour at *JXB* online.)

Collectively, these results indicate that NH_4_^+^ nutrition provokes mild chronic stress that leads to the activation of SAA responses that prime tomato defence pathways and induce resistance against subsequent biotic stress. NH_4_^+^ nutrition enhanced H_2_O_2_ accumulation, which acts as a signal to induce ABA-dependent responses, reducing the NH_4_^+^ toxicity. These ABA-dependent signalling pathways induce PA biosynthesis and catabolism, which in turn enhance H_2_O_2_ accumulation, favouring stress signal amplification. Moreover, NH_4_^+^ nutrition induces Put accumulation, and it was demonstrated that compromising Put accumulation provokes increased susceptibility to *Pst*. Conversely, the basal stomatal closure observed in N-NH_4_^+^ plants was probably produced by H_2_O_2_ derived from enhanced CuAO activity induced by ABA-mediated NH_4_^+^ responses. The basal stomatal closure observed in NH_4_^+^ plants may reduce the entry of bacteria into the mesophyll, reducing the diseased symptoms, favouring the NH_4_^+^-IR. Besides these functions, it is speculated that H_2_O_2_ acts as a signal of NH_4_^+^ stress and primes plant defence responses against subsequent pathogen infection. Although classical SA-dependent responses against biotrophic pathogens were not found, other defence mechanisms were observed related to the resistance of these lifestyle pathogens, namely strong and fast oxidative bursts, probably mediated by induction of *rboh1* and *CuAO* genes, which directly reduce the spread of pathogens in the plant (Fig. 9). The study of the mechanism of action of NH_4_^+^ as an inducer of resistance showed that N-NH_4_^+^ plants displayed basal responses related to the establishment of the acclimation state in systemic leaves through SAA-related mechanisms. This activation allows the plant to trigger more efficient and effective specific responses to prevent pathogen spread, and therefore induce resistance to disease. The NH_4_^+^-IR mediated by SAA mechanisms is especially important because it has been shown to be effective in other species against abiotic stress (Fernandez-Crespo *et al.*, 2012) and against two different lifestyle pathogens, *Pst* and *B. cinerea*. The study of events underlying this kind of induced resistance, which is effective against biotic and abiotic stress, provides knowledge necessary to exploit this phenomenon in the context of sustainable agriculture.

## Supplementary data

Supplementary data are available at *JXB* online.

Figure S1. Spm and Spd content in control and N-NH_4_^+^ tomato plants during *Pst* infection.

Figure S2. Effect of NH_4_^+^ treatment on tomato plants infected with *B. cinerea*.

Table S1. Primer sequences.

Supplementary Data
