# Supplementary material for: NH4 + protects tomato plants against Pseudomonas syringae by activation of systemic acquired acclimation
Source: J Exp Bot. 2015 Aug 5;66(21):6777–90. doi: 10.1093/jxb/erv382 (PMC4623687; doi:10.1093/jxb/erv382)
Supplement: Supplementary Data [file supp_66_21_6777__index.html]

NH4+ protects tomato plants against Pseudomonas syringae by activation of systemic acquired acclimation — NH4 + protects tomato plants against Pseudomonas syringae by activation of systemic acquired acclimation — Supplementary Data 

# NH4+ protects tomato plants against *Pseudomonas syringae* by activation of systemic acquired acclimation

## Supplementary Data

Data files

- Supplementary Data - Supplementary Data
