# Supplementary material for: NH4 + protects tomato plants against Pseudomonas syringae by activation of systemic acquired acclimation
Source: J Exp Bot. 2015 Aug 5;66(21):6777–90. doi: 10.1093/jxb/erv382 (PMC4623687; doi:10.1093/jxb/erv382)
Supplement: Supplementary Data [file supp_erv382_jexbot150151_file001.pdf]

NH_4^+ protects tomato plants against Pseudomonas syringae by activation of systemic acquired acclimation

Emma Fernández-Crespo, Loredana Scalschi, Eugenio Llorens , Pilar García-Agustín, and Gemma Camañes

Supplemental Files

Table S1. Primers sequences

Gene	GenBank accession number	Tm	Primers
EF1α	NM_001247106.1	60	Fwd: 5′-GACAGGCGTTCAGGTAAGGA-3′<br>Rev: 5′ -GGGTATTCAGCAAAGGTCTC-3′
PR1	EU589238.1	54	Fwd: 5′-CCGTGCAATTGTGGGTGTC-3′<br>Rev: 5′-GAGTTGCGCCAGACTACTTGAGT-3′
PR5	AY257487.1	58	Fwd: 5′-GAGGTTCATGCCAAACTGGTC-3′<br>Rev: 5′-TCAACCAAAGAAATGTCC-3′
LoxD	U37840.1	58	Fwd: 5′-GGCTTGCTTTACTCCTGGTC-3′<br>Rev: 5′-AAATCAAAGCGCCAGTTCTT-3′
ACCox	NP_001233928.1	54	Fwd: 5′-CCATGTCCTAAGCCCGATTTGAT-3′<br>Rev: 5′-TCACTTT GTCATCTTGGAACAGA-3′
JMT	Scalschi et al., 2013	55	Fwd: 5′-GGTTCAAAGTGCATGAGAGCT-3′<br>Rev: 5′-TACACCACACACTGAAGGAAA-3′
Asr1	NM_001247703.1	54	Fwd: 5′-ACACCACCACCACCTGT-3′<br>Rev: 5′-GTGTTTGTGTGCATGTTGTGGA-3′
rboh1	AF148534.1	60	Fwd: 5′-AAGCCGGAACTTGAGTCAGA-3′<br>Rev: 5′-GTCTCAGCAGCACCCTTAGC-3′
CuAO	AJ871578.1	52	Fwd: 5′-ATATCCCGGGTCATGTTTGA-3′<br>Rev: 5′-TCCATCAGGTGCCACAAATA-3′

Fig.S1. PAs content in control and N-NH_4^+ tomato plants in *Pst* infection. Four-week-old tomato plants were grown in control conditions or in 5 mM of NH_4^+ and were inoculated by dipping them in a bacterial suspension of *Pst* at 5×10^5 c.f.u. mL^{-1}. Leaves were collected at various time points and spermidine (A) and spermine (B) levels were determined in freeze-dried material by HPLC–MS. Data show the average of three independent experiments of a pool of 10 plants per experiment ± SE. Letters indicate statistically significant differences ($p < 0.05$; least-significant difference test).

Fig.S2. Effect of NH_4^+ treatment on tomato plants infected with *B. cinerea*. Four-week-old tomato plants were grown in control conditions or in 5 mM of NH_4^+ and lesion diameter was measured after 72 h of inoculation. The data show the lesion diameter (mm) ± standard error (SE) (n = 20)

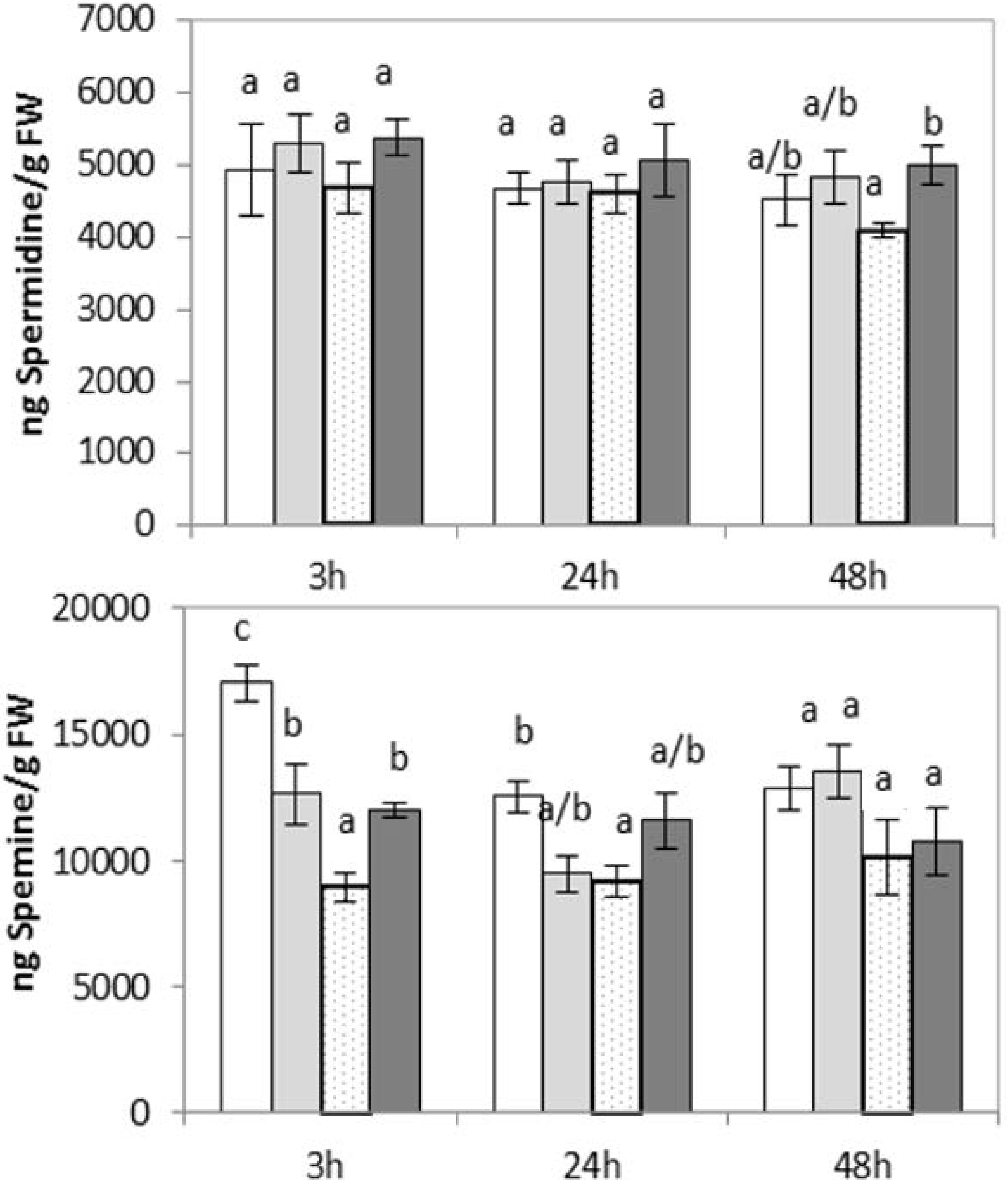
7000
6000
5000
4000
3000
2000
1000
0
ng Spermidine/g FW
a
a
a
a
a
a
a
a
a/b
a/b
a
b
3h
24h
48h
20000
15000
10000
5000
0
ng Spemine/g FW
c
b
a
b
b
a/b
a
a/b
a
a
a
a
3h
24h
48h

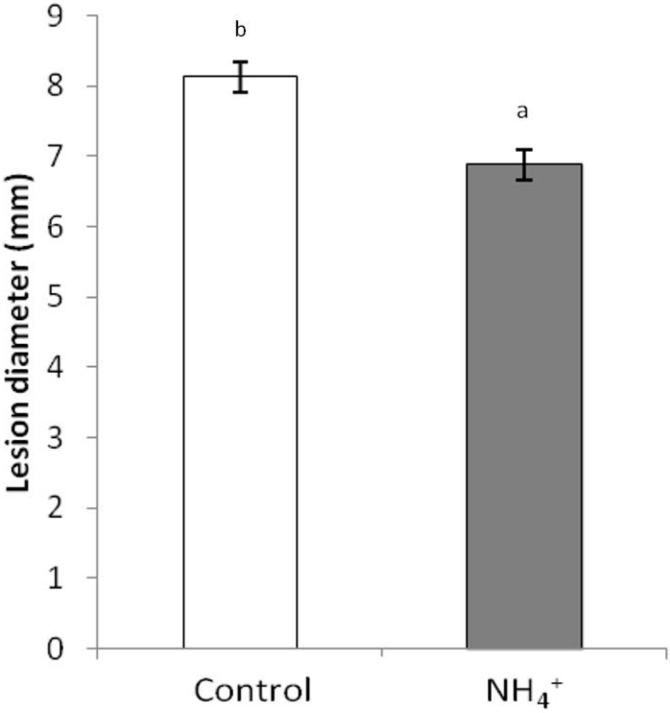

Lesion diameter (mm)
9
8
7
6
5
4
3
2
1
0
b
a
Control
NH_4^+
